# Non-alcoholic Fatty Liver Disease and Carbohydrate Restricted Diets: A Case Report and Literature Review

**DOI:** 10.7759/cureus.18641

**Published:** 2021-10-10

**Authors:** Luxhman Gunaseelan, Umna S Khan, Fatima Khalid, Muhammad A Hamid

**Affiliations:** 1 Medicine, Saba University School of Medicine, Toronto, CAN; 2 Medicine and Surgery, Dow International Medical College, Karachi, PAK; 3 Medicine and Surgery, University College of Medicine and Dentistry, Lahore, PAK; 4 Pediatrics, University of Toronto, Toronto, CAN

**Keywords:** young male, south asian, nafld, non-alcoholic fatty liver disease, non-alcoholic steatohepatitis, carbohydrate-restricted diet, low-carbohydrate diet

## Abstract

Non-alcoholic fatty liver disease is the accumulation of excessive fat in the liver. Various treatment options are available to manage the condition, among which carbohydrate restriction has been shown to reduce liver fat accumulation, liver inflammation, serum liver enzyme levels, and hepatic de-novo lipogenesis in people with non-alcoholic fatty liver disease. Here, we present a case report of a 25-year-old South Asian patient presenting with right upper quadrant pain, fatigue, and headaches. After confirmation of non-Alcoholic fatty liver disease (NAFLD) diagnosis by biopsy, the patient initiated a low-carbohydrate diet. Four months after which significant improvement was noticed in clinical and laboratory parameters. Peer-reviewed publications were then sourced from online databases to explore the efficacy of low-carbohydrate diets for NAFLD. Our results were compared with the existing data. However, limited literature existed for such an intervention in the South Asian population therefore, the case report is novel. Combined with findings from the literature, our results from the case report supported our hypothesis that carbohydrate restriction might promote a reduction in hepatic fat accumulation and inflammation in patients with NAFLD and diabetes in various ethnicities including South Asians.

## Introduction

Non-alcoholic fatty liver disease (NAFLD) is defined as the accumulation of excess liver fat with unknown etiology [1]. The pathophysiology of NAFLD can be described as increased storage and synthesis of fat, and decreased elimination of fat, in liver cells, such as through impaired beta-oxidation and lipid export [1]. NAFLD is an array of clinicopathological features ranging from a stage as simple as steatosis, which is simply the asymptomatic accumulation of fat without inflammation to cirrhosis where liver function is deranged and symptoms such as jaundice start to manifest [1]. 

NAFLD is strongly associated with metabolic syndrome - a clustering of metabolic abnormalities associated with insulin resistance, particularly in obese sedentary patients [2]. Metabolic syndrome is signified by the presence of at least three of the following: type 2 diabetes, hypertriglyceridemia, low high-density lipoprotein (HDL) cholesterol, increased waist circumference, and hypertension [3]. Type 2 diabetes is seen in 30-50% of all NAFLD patients and 97% of NAFLD patients are overweight or obese [1]. Obesity induces selective insulin resistance in adipose tissue and hyperinsulinemia in liver cells, thereby causing NAFLD. Insulin resistance is characterized by reduced levels of whole-body, hepatic and adipose tissue sensitivity to insulin. Obesity, metabolic syndrome, and insulin resistance act as the first hit of the two-hit hypothesis in the development of NAFLD followed by the second hit which is oxidative stress.

Other risk factors for developing NAFLD include abdominal surgeries namely bariatric surgeries, jejunal bypass, small bowel resection and biliopancreatic diversion, periods of acute starvation, total parenteral nutrition, and some drugs such as amiodarone, tamoxifen, glucocorticoids, and synthetic estrogens [1]. Treatments for NAFLD target liver inflammation and hepatocyte fat accumulation. Inhibitors of inflammatory cytokines, such as infliximab, a tumor necrosis factor-alpha (TNF-alpha) inhibitor, target liver inflammation. Infliximab reduces liver inflammation by decreasing the production of the pro-inflammatory molecules TNF-alpha, interleukin-6 (IL-6), interleukin-1 (IL-1), and suppressor of cytokine signaling-3 (SOCS-3) [4]. 

NAFLD treatments that target hepatocyte fat accumulation include pharmacotherapy, dietary modification, and interventions that promote weight loss. Weight loss is achieved through exercise, calorie restriction, bariatric surgery, and the use of drugs such as orlistat and sibutramine, which inhibit dietary fat absorption in the gut. Regular exercise is effective in treating NAFLD by reducing visceral fat, subsequently decreasing insulin resistance and liver fat accumulation. It also enables the channeling of dietary carbohydrates to muscle glycogen instead of the liver for lipogenesis [5]. 

Pharmacotherapy has shown to be effective in reducing insulin resistance by improving gluconeogenesis, glycolysis, and peripheral glucose uptake with drugs like metformin [6]. Dietary modification, such as a diet low in simple carbohydrates, is also highly effective in treating NAFLD. A low-carbohydrate (LC) diet is one in which less than 40% of total calories come from carbohydrates. A high-carbohydrate (HC) diet is one in which more than 50% of total calories come from carbohydrates [1]. 

LC diets also reduce blood glucose levels. This in turn reduces blood insulin levels, thereby decreasing hepatocyte lipogenic enzyme formation, which occurs when insulin binds to sterol receptor binding protein 1c [6]. Lower blood glucose levels also provide less substrate for liver lipogenesis. Carbohydrate restriction forces hepatocytes to rely more on energy generated outside of glucose metabolism, thereby promoting hepatic beta-oxidation, and eventually hepatic fat reduction [7]. Carbohydrate restriction also results in decreased liver inflammation, as carbohydrates stimulate the synthesis and release of TNF-alpha in liver cells by upregulating sterol regulatory element-binding protein-1c, which promotes liver inflammation [6].

NAFLD patients coming from different ethnic backgrounds have been shown to respond to dietary interventions. The South Asian population is highly susceptible to NAFLD but there is limited data available on the effectiveness of carbohydrate restriction in treating NAFLD in this population. This case report provides evidence of the positive impact of low carbohydrate diet intervention on hepatic fat, hepatic inflammation, and serum liver enzymes in a young South Asian male. 

A literature review was also conducted to investigate the effect of carbohydrate restriction on NAFLD through its influence on liver fat accumulation, liver inflammation, and serum liver enzyme levels: Alanine transaminase (ALT) and Aspartate aminotransferase (AST). Randomized clinical trials and case-control studies were predominantly used to evaluate the evidence for the efficacy of an LC diet to ameliorate NAFLD, while observational studies were considered in terms of supporting evidence. 

## Case presentation

We present a case of a 25-year-old South Asian male with no past medical history who was seen for evaluation of a six-month history of right upper quadrant pain associated with fatigue and headaches. The patient denied nausea, changes in weight, or jaundice. He had no evidence of diabetes mellitus, or hypercholesterolemia, although he was overweight, with a body mass index (BMI) of 27.8 kg/m2. Medication history, prescribed or over the counter, was negative. Physical exam revealed hepatomegaly (liver span = 21 cm). Laboratory findings (Table 1) revealed elevated concentrations of serum alanine aminotransferase (ALT) 155 U/L and aspartate aminotransferase (AST) 59 U/L. A full workup was conducted to rule out other causes of elevated liver enzymes. Autoimmune and viral serologies were negative. Serum thyroid-stimulating hormone (TSH) and iron levels were unrevealing. Helicobacter pylori stool antigen tests were negative. The patient abstained from alcohol, was not taking any medication, and did not have a known exposure to other environmental causes of liver disease. The patient had no family history of diabetes, hyperlipidemia, or liver disease.

**Table 1:** Diagnostic testing performed to rule out other causes of elevated liver enzymes, fatigue, or right upper quadrant abdominal pain. ALT- Alanine Aminotransferase, AST - Aspartate Aminotransferase, HbA1C - Glycated Hemoglobin, HDL- High-Density Lipoprotein, LDL - Low-Density Lipoprotein, TIBC - Total Iron Binding Capacity.

Diagnostic Lab Testing	Initial Visit	2 months	6 months
Liver Function Test			
ALT (U/L)	155	138	36
AST (U/L)	59	54	30
HbA1C	4.50%	-	-
Lipid Panel			
Total Cholesterol (mg/dL)	182	-	-
HDL (mg/dL)	43.5	-	-
LDL (mg/dL)	99.2	-	-
Triglycerides (mg/dL)	197	-	-
Anti-Hepatitis C antibody	Negative	-	-
Hepatitis A IgG antibody	Negative	-	-
Hepatitis B			
Surface antigen	Negative	-	-
Surface antibody	Positive	-	-
Core antibody	Negative	-	-
Iron Studies			
Iron	105mcg/dL	-	-
Ferritin	234ng/mL	-	-
TIBC	280mcg/dL	-	-
Serum Gammaglobulin	Negative	-	-
Antinuclear antibody (ANA)	Negative	-	-
Anti-smooth muscle antibody	Negative	-	-
Anti-liver/Kidney microsomal antibody-1	Negative	-	-
Thyroid Stimulating Hormone	4.4	-	-
H-Pylori stool antigen test	Negative	-	-

Ultrasound examination revealed no gallstones in the gallbladder or biliary tree abnormalities. The liver, however, had a hyperechoic texture, consistent with diffuse fatty infiltration. Liver biopsy revealed steatosis with lobular inflammation and hepatocyte ballooning without fibrosis, consistent with a diagnosis of non-alcoholic steatohepatitis. 

An exercise and calorie restriction diet regimen of 1800 kcal/day was initiated to promote weight loss. His exercise regimen consisted of moderate-weight strength training and jogging for one hour three times per week. At two months follow up, despite a BMI reduction to 26.5 kg/m2, the patient’s symptoms of right upper quadrant abdominal pain, headache, and fatigue still persisted. Liver enzymes still remained elevated (ALT= 138 U/L, AST=54 U/L). In light of literature documenting the efficacy of LC diets for fatty liver disease, the calorie restriction diet regimen was switched to the Atkins diet, a popular LC diet. Dietary composition over the next three months was 50% protein, 30% fat (predominantly polyunsaturated and monounsaturated fat), and 20% carbohydrates, with an estimated caloric intake of 2000 kcal. Foods containing complex carbohydrates, such as beans, legumes, and whole grains, were the predominant source of carbohydrates, and large amounts of leafy green vegetables were consumed in the form of raw salads to ensure sufficient fiber intake. 

Four months after the initiation of a low carbohydrate diet, at a follow-up, the patient’s BMI had fallen to 25.5 kg/m2. The patient reported significant improvement of his right upper quadrant abdominal pain, fatigue, and headache. His serum liver enzymes were normal (ALT= 36 U/L, AST=30 U/L). Repeat ultrasound revealed mild liver echogenicity, significantly improved from previous imaging. The patient was counseled to continue the same diet and return to the clinic if symptoms recurred.

## Discussion

To put our case in context, the PubMed database was used to identify eight studies on LC diets and NAFLD (Table 2). These studies considered a total of 444 study participants, including 69 patients with NAFLD, 311 patients with diabetes or insulin resistance, and 382 patients with obesity. In addition to patients, this review includes results for 32 healthy, non-obese controls, and data from 8,964 individuals without prior diagnosis of diabetes or liver disease, obtained from the Japanese Ministry of Health database. The studies included subjects in a wide age range: 18-65 years. The age range of all patients was 9-65 years, the age range of patients with diabetes was 30-65 years, the age range of patients with obesity was 24-64 years, and the age range of patients with NAFLD was 18-65 years. 

**Table 2:** Studies investigating the impact of low carbohydrate dietary interventions on the development of Non-Alcoholic Fatty Liver Disease (NAFLD)

Study	Study Design	Characteristics of Patients/Controls	Age Range	Ethnicity	Dietary Interventions	Results
Fraser et al., 2008 [8]	Quasi-randomized controlled trial (allocation by alternation)	259 participants with obesity and type 2 diabetes	30-65	not mentioned	American Diabetes Association Diet: n=85 Low Glycemic Index diet: n=89 Modified Mediterranean Diet: n=85	Change in serum after 12 months: ALT levels: ADA diet: 5.2 (P<0.001) LGI diet: 4.8 (P<0.001) MM diet: 10.1 (P<0.001)
Browning et al., 2011 [7]	Randomized controlled trial	18 participants with obesity and NAFLD	31-59	not mentioned	Low Calorie Diet: n=9 Low Carbohydrate Diet: n=9	Change in hepatic triglyceride content after 2 weeks: Low Calorie Diet: (-28 ± 23%) (P<0.004) Low Carbohydrate Diet: (-55 ± 14%) (P<0.004)
Ryan et al., 2007 [9]	Post hoc analysis of randomized controlled trial	52 obese patients	38-64	92% caucasians in 60% carbohydrate diet group; 93% caucasians in 40% carbohydrate diet group	60% Carbohydrate Diet: n=26 40% Carbohydrate Diet: N=26	Change in serum ALT after 16 weeks: 60% carbohydrate diet: 9.5 units/L (P<0.01) 40% carbohydrate diet: 4.1 units/L (P<0.04)
Martens et al., 2014 [10]	Single-blinded, randomized, parallel group superiority trial	16 healthy participants;	19-29	not mentioned	High Protein Low Carbohydrate diet: n=9 High Carbohydrate Low Protein diet: n=7	Change in hepatic triglyceride content after 12 weeks: HPLC: IHTG%: 0.25±0.20% to 0.22±0.11% HCLP: IHTG%: 0.38±0.22% to 0.43±0.24%
Toshimitsu et al., 2007 [11]	Case Control Study	46 study participants, 8964 healthy controls	18-65	not mentioned	NASH: n=28 Fatty Liver: n= 18 Healthy Controls: n= 8964	Mean Carbohydrate Intake: NASH: 406 ±176 kcal FL: 409 ±90 kcal Controls: 273 ±89 kcal % Simple Carbohydrate intake: NASH: 6.63 ±4% FL: 5.1 ±3.6% Controls: 2.77 ±0.97 % Mean Calorie Intake: NASH: 2790 ±1134 kcal FL: 3179 ±775 kcal Controls: 1942 ±603 kcal
Sevastinova et al., 2012 [12]	Crossover Trial	16 obese volunteers	age <18	not mentioned	High Carbohydrate Diet for 3 weeks followed by Low Carbohydrate Diet for 6 months	After 3 weeks on the high carbohydrate diet, participants gained 1.8±0.3 kg (P<0.0001) and had 2.5±1.9% increase in liver fat content (P<0.005) After 6 months on the low carbohydrate diet, participants lost 3.2±0.6 kg (P<0.0001) and had 2.9±1.9% decrease in liver fat content (P<0.05)
Amy et al, 2020 [13]	Randomized two arm parallel dietary intervention	32 participants with obesity and NAFLD	Sep-17	White: n=23; Hispanic:n=8; Asian: n=1	Carbohydrate restricted diet (CRD): n=16; Fat restricted diet (FRD): n=16	8 weeks after intervention, participants on CRD lost 5.5% total fat mass, 32% hepatic lipid content. A decline in ALT, AST, fasting insulin and insulin resistance
Tendler et al., 2007 [14]	Prospective single arm clinical pilot trial	5 obese participants (mean BMI= 36.8 kg/m2)	18-65	not mentioned	6-month low carbohydrate diet (<20g carbohydrates/day)	Changes after 6 months: Mean % change in weight: -10.9% (P=0.036) Reduction in hepatic steatosis (P=0.02) Reduction in liver inflammation (P=0.05)

**Figure 1:**
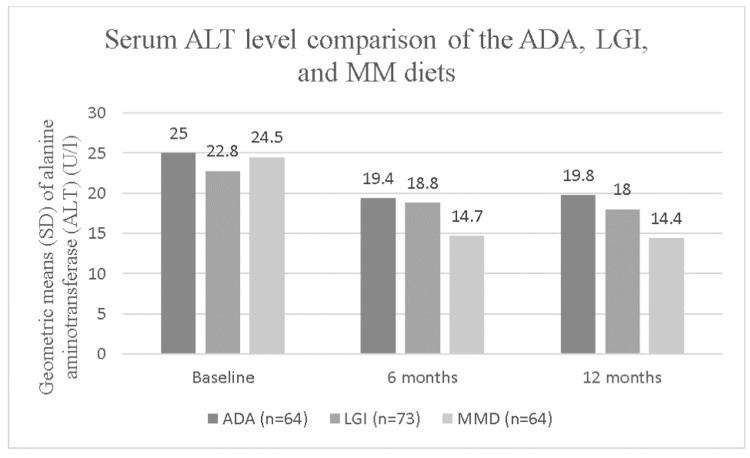
Serum alanine aminotransferase (ALT) level comparison between the American Diabetes Association (ADA), low glycemic index (LGI), and modified Mediterranean (MM) diets. The MM diet had the greatest 12-month improvement of serum ALT levels (10.1, p <0.001) among the three; approximately twice the improvement of the LGI (4.8, p<0.001) and the ADA (5.2, p<0.001) diets. Adapted from Fraser et al., 2008.

In the first study considered, a quasi-randomized controlled trial of 259 obese, diabetic participants (allocation by alternation), Fraser et al. [8] investigated the effect of an LC diet on serum liver enzyme levels. The study used photometric methods to evaluate the effect of different dietary interventions on the patients’ ALT levels [8]. Participants were allocated using systematic sequence to one of three diets which were similar in total energy intake: the 2003 American Diabetes Association (ADA) diet (50-55% carbohydrates, 30% fat, and 15-20% protein), a low glycemic index (LGI) diet (50-55% LGI carbohydrates, 30% fat, and 15-20% protein), and a modified Mediterranean diet (35% LGI carbohydrates, 45% fat, mostly monounsaturated, and 15-20% protein) [8]. The recruiter and participants were both blinded to the allocation procedure. The ADA diet included whole wheat bread, whole grain rice, bran flakes, rice cakes, fruit, vegetables, low-sugar jam, and honey. The LGI diet replaced bran flakes with oats, and bread and rice with lentils, chickpeas, and white beans. The Mediterranean diet included fish, replaced butter with olive oil and olive-based margarine, and included almonds and other nuts. Each diet included egg, chicken, and turkey breast. Of the 259 participants, 85 followed the ADA diet, 89 followed the LGI diet, and 85 followed the Mediterranean diet. Of the original 259 participants, 58 participants did not follow up at 6 months, and 22 more were lost to follow up at 12 months. At 6-months, the LC modified Mediterranean diet had the greatest effect in lowering ALT levels (Figure 1), and at one year, the modified Mediterranean diet showed the greatest improvement, with a serum ALT decrease of 10.1 (P<0.001) at 12-month follow-up. The LGI diet improved ALT levels the least, with a one-year serum ALT decrease of only 4.8 (P<0.001). The ADA diet had a one-year serum ALT decrease of 5.2 (P<0.001). Most of the change in ALT occurred over the first 6 months, with only slight changes thereafter: 6 months of the modified Mediterranean diet resulted in a serum ALT decrease of 9.8 (P<0.001), with a further decrease of 0.3 by 12 months (P<0.001). With the ADA diet, ALT increased by 0.4 points (P<0.001) between 6 and 12 months. With the LGI diet, ALT decreased by 0.8 points (P<0.001) between 6 and 12 months.

Ryan et al. [9] conducted a post hoc analysis of 52 obese individuals with insulin resistance and normal serum ALT levels (< 60 units/l). Participants were randomly allocated to one of two hypocaloric (similar in total energy intake) diets - a low-carbohydrate diet (LC; 40% carbohydrates, 45% fat, and 15% protein) or a high carbohydrate diet (HC; 60% carbohydrates, 25% fat, and 15% protein) [9]. Only complex carbohydrates were utilized in the diets, and study participants in both groups were encouraged to consume a minimum of 25 grams of fiber daily. Weight and serum ALT levels were obtained at baseline and after 16 weeks of dieting. The LC diet had the greatest effect in lowering serum ALT levels after 16 weeks [9], with a reduction of 9.5 units/L (P < 0.01), compared with the HC diet, which lowered ALT by 4.1 units/L (P < 0.04). 

To evaluate the effect of different dietary interventions on intrahepatic triglyceride content in healthy participants, Martens et al. [10] conducted a single-blinded, randomized, parallel-group superiority trial with 16 healthy participants. Participants were randomly allocated to one of two diets that were similar in total energy intake: a high-protein, LC diet (HPLC; 30% carbohydrates, 35% fat, and 35% protein) or an HC, low-protein diet (HCLP) with 60% carbohydrates, 35% fat, and 5% protein [10]. Whether the carbohydrates were simple or complex was not specified. Serum intrahepatic triglyceride levels were obtained at baseline and after 12 weeks. This was a single-blind study; potential participants were blinded to the allocation procedure and its outcome. Participants were given dietary guidelines for both the HPLC diet and the HCLP diet, recipes for each diet, whey protein supplements for the HPLC dieters, and maltodextrin for the HCLP dieters. Nine participants completed the HPLC diet and seven successfully completed the HCLP diet. Hepatic triglyceride content was assessed using proton magnetic resonance spectroscopy and MRI. After 12 weeks of dieting, the HPLC diet had the greatest effect in lowering hepatic triglyceride levels, which decreased from 0.25% to 0.22% [10]. The HCLP diet, in contrast, increased intrahepatic triglyceride content, from 0.38% to 0.43%. These results suggest that an HPLC diet may lower hepatic steatosis and that an HCLP diet may increase steatosis (p = 0.055). Bodyweight and BMI did not change significantly in either group. 

Browning et al. [7] conducted a randomized controlled trial with 18 NAFLD patients to evaluate the effect of a two-week carbohydrate-restricted diet versus a calorie-restricted diet on weight loss and intrahepatic triglyceride content in NAFLD patients. Participants were randomly allocated to either a carbohydrate-restricted diet (less than 20 g/day of carbohydrates) or a low-calorie diet (maximum of 1500 kcal/day). All patients had maintained a stable weight for the past 6 months and none were currently enrolled in weight loss programs [7]. Subjects were first weighed and then underwent magnetic resonance spectroscopy to determine baseline weight and liver steatosis. They adhered to their assigned diet for two weeks. For the first week, participants were allowed to choose their meals, as long as they met the dietary requirements. Frozen meals that adhered to the dietary guidelines for each group were provided during the second week. The study did not specify whether the carbohydrates in the diets were simple or complex. After the diet, patients were weighed again and magnetic resonance spectroscopy was used to determine liver steatosis. Intrahepatic triglyceride content decreased more in the LC diet group (-55 ± 14%) than in the low-calorie diet group (-28 ± 23%) [7]. 

Toshimitsu et al. [11] used an observational case-control method to assess the dietary habits and nutrient intake of 28 patients with NASH and 18 patients with simple steatosis. The NAFLD patient data were also compared with survey data from 8964 randomly selected healthy controls, obtained from the Japanese Ministry of Health, Labor, and Welfare. Participants were sorted into three age groups: 20-39, 40-59, and over 60 [11]. For three consecutive days, the patients recorded their dietary habits and nutrient intake, with the help of dietitians. Carbohydrate, protein, fat (polyunsaturated, monounsaturated, and saturated), and zinc intake were monitored. Serum liver enzyme levels were determined for each patient, and NASH or simple steatosis was confirmed by needle biopsy. Patients with NASH and fatty liver consumed far more dietary carbohydrates than healthy individuals. Randomly selected healthy individuals consumed less than 280 grams of carbohydrates per day while those with NASH and fatty liver consumed far more. This trend is seen especially in the 20 to 39-year-old group with NASH, who consumed roughly 508 grams of carbohydrates per day (simple carbohydrates comprising 6.2% of total carbohydrates), compared with their healthy peers, who consumed only 265 grams (simple carbohydrates comprising 2.5% of total carbohydrates). Total calorie intake was also much higher in NASH and NAFLD patients, in all age groups, but especially in the 20-39 age group; NASH and NAFLD patients in this age range consumed approximately 3700 kcal per day while their healthy counterparts consumed only 2000 kcal [11]. These results suggest that an HC and high-calorie diet may increase hepatic steatosis and NASH development16. 

Sevastianova et al. [12] conducted a crossover trial with 16 obese volunteers to investigate the effects of an HC diet on hepatic steatosis and de-novo lipogenesis, and the efficacy of an LC diet in treating NAFLD by reducing hepatocyte steatosis and de-novo lipogenesis. Participants were negative for alcoholic fatty liver disease, viral hepatitis, pregnancy, and the PNPLA3 genotype, which predisposes individuals to increase liver fat accumulation. After obtaining baseline weight, baseline liver fat was measured through magnetic resonance spectroscopy [12]. De-novo lipogenesis was assessed by the ratio of serum short-chain fatty acids to medium-chain fatty acids; a higher ratio of short- to medium-chain fatty acids indicates increased de-novo lipogenesis. Participants then underwent a three-week HC diet (> 1000 kcal of simple carbohydrates daily), under the consultation of a dietitian. Measurements were taken again after the carbohydrate overconsumption. Participants then switched to an LC diet for six months and were measured again. The HC diet resulted in increased weight gain (1.8 kg) and de-novo lipogenesis (a 50% increase in the ratio of short-chain to medium-chain fatty acids in serum triglycerides, and a 120% increase in short-chain to medium-chain fatty acids in serum VLDL triglycerides). The LC diet resulted in weight loss (3.2 kg). Consumption of a diet high in simple carbohydrates increased intrahepatic triglyceride content, and subsequent consumption of an LC diet for six months decreased intrahepatic triglyceride content. 

Similar to the above-mentioned study, Amy et al. [13] conducted an eight-week randomized two-arm, parallel dietary intervention to compare the effects of carbohydrate-restricted diet (CRD) and fat-restricted diet (FRD) on hepatic fat content in adolescents (age range: 9-17 years) with NAFLD and obesity.

The 32 participants were from multiple ethnic backgrounds (White, Hispanic, and Asian). Baseline investigations included fasting lipid profile, fasting liver enzymes, blood glucose, insulin levels. Weight, height, BMI, and hepatic fat fraction (HFF) were also recorded. Hepatic fat was evaluated using a 3-point M Dixon MRI while body composition was determined using dual-energy X-ray absorption (DXA). An equal number of participants were randomly assigned to either CRD (≤ 25% energy from CHO, 25% energy from protein, and ≥ 50% energy from fat) or FRD (55:25:20% energy from CHO:protein:fat), which they adhered to for eight weeks under the counseling of a registered dietician. Only the personnel studying the outcomes were blinded to the group assignments. The modified feeding phase continued for the first two weeks, where groceries were provided to the families. All the investigations and measurements were repeated at the end of the intervention where individuals on CRD lost more BMI, BMI z score, total body fat. There was also a decline in serum ALT, AST, fasting insulin, and insulin resistance. A considerable reduction was noted in hepatic lipid content (32%) within the CRD group. One limitation to the study is that liver fat content can not be assessed independently of body fat loss since participants in the CRD group experienced weight loss despite providing a diet aiming to keep weight constant.

Tendler et al. [14] conducted a prospective single-arm clinical pilot trial of five obese patients (mean BMI = 36.8 kg/m2) with NAFLD (diagnosed by liver biopsy) to investigate the effects of an LC diet on hepatic steatosis, inflammation, fibrosis, and body weight. A pre-study liver biopsy was conducted to establish baseline hepatic steatosis, fibrosis, and inflammation. All patients either abstained from alcohol or had a maximum of five alcoholic beverages annually [14]. Patients adhered to an LC diet for six months. They were instructed to consume a maximum of < 20 g/d of either simple or complex carbohydrates daily. The initial diet consisted of unlimited amounts of meat, eggs, and LC vegetables. Caloric intake was not restricted. Diet compliance was assessed through urinary ketone strips. At the end of the diet, serum liver enzymes and body weight were measured, and another liver biopsy was conducted to measure final hepatic steatosis levels. Hepatocyte fibrosis was graded according to the density of collagen fibrotic tissue in the liver. During the six-month LC diet, four out of five patients experienced a mean weight loss of 12.8 kg compared with baseline weight, with a mean percentage change in weight of -10.9% (P = 0.036). These patients also showed reduced hepatic steatosis (P = 0.02), inflammation (P = 0.05), and fibrosis (P = 0.07). One patient did not lose any weight, and liver histology actually became worse. However, this patient had many negative urinary ketone tests and only one positive urinary ketone test, suggesting failure to adhere to the diet. 

Our case and the above-described studies suggest that carbohydrate restriction could be an effective way in the management of NAFLD. The two-hit hypothesis explains the pathophysiology behind the consumption of a high carbohydrate diet and the development of NAFLD. In hit 1 of the 2-hit NAFLD hypothesis, due to insulin resistance hepatocytes become less responsive to insulin and excess insulin in hepatocytes binds to sterol receptor binding protein (SREBP-1c), stimulating the synthesis of lipogenic enzymes, which promote the synthesis and accumulation of fat in the liver cells [1]. Because there is decreased hepatic insulin sensitivity, insulin is no longer able to control the production of glucose and very-low-density lipoprotein (VLDL) thereby, promoting gluconeogenesis and lipogenesis [15]. In addition, consumption of excessive carbohydrates results in increased hepatic de-novo lipogenesis as evident by Sevastinova et al. [12] study where the participants were overfed with simple carbohydrates resulting in accelerated hepatic de-novo lipogenesis. On the contrary, Tendler et al. [14], Browning et al. [7], Martens et al. [10], and Amy et al [13], all found that an LC diet lowered hepatic triglyceride content more than a low-calorie diet. Browning et al. [7] found that the LC diet decreased intrahepatic triglyceride content by 12%, compared with 5% in the low-calorie HC diet. Marten et al. [10] found that a 12-week HC, low-protein diet promoted intrahepatic triglyceride content formation by 0.05%, while a 12-week high-protein, LC diet reduced intrahepatic triglyceride content by 0.03%, while Amy et al reported a drop of 32% in hepatic lipid content with carbohydrate-restricted diet group compared to fat-restricted diet group. Comparing our case report with the aforementioned studies reveals that the results were consistent. After initiating an LC in our patient, there was a decline in intrahepatic fat content as evident by improvement in liver echogenicity on repeat ultrasound.

In hit 2 of the two-hit NAFLD hypothesis, beta-oxidation of excessive hepatic fat results in inflammation and fibrosis. This depletes glutathione and vitamin E, thereby promoting oxidative stress, which results in lipid peroxidation and release of three inflammatory cytokines: tumor necrosis factor-alpha (TNF-alpha), interleukin 1 (IL-1), and interleukin 6 (IL-6) [1]. TNF-alpha induces inflammation and increases insulin resistance by downregulating adiponectin. Adiponectin decreases liver inflammation by downregulating aldehyde oxidase 1, a reactive oxygen species producer. Adiponectin also increases insulin sensitivity and decreases insulin secretion, thereby lowering liver fat accumulation [4].) These inflammatory cytokines also promote liver inflammation and injury to the liver parenchyma, leading to increased liver collagen deposition and eventual fibrosis, as noted in Toshimitsu et al. [11]. A limitation of this observational study, however, is its reliance on 72-hour diet recall surveys to record nutrient intake, making it very susceptible to recall bias. A 72-hour diet recall survey also does not provide comprehensive information about a person’s general eating habits. At best, this study is useful in identifying trends in carbohydrate intake and NAFLD development. 

The outcomes stemming from the dietary intervention in our case also include a meaningful improvement in serum ALT (declined from 155 U/L at the initial visit to 36 U/L at 6 months follow up visit) and AST (declined from 59 U/L at the initial visit to 30 U/L at 6 months follow up) after consuming LC diet which is accordant with the results of studies conducted by Fraser et al. [8], and Ryan et al [9] and Amy et al [13], who note a reduction in serum ALT levels with an LC diet. Fraser et al. [8] show that an LC diet, such as the modified Mediterranean diet, lowers serum ALT levels more than the standard American diet and the low glycemic index diet. Of note, the low glycemic index diet fared comparably to the ADA diet, even though according to Pepa et al. [16], higher glycemic index foods promote hepatic steatosis by increasing insulin resistance through hyperglycemia. 

Fraser et al. [8] and Ryan et al. [9] also show that both low-calorie and LC diets lower serum ALT by promoting weight loss, although it must be noted that the LC diet was far more effective in lowering serum ALT, despite being eucaloric to the low-calorie diet. Keeping in view the literature review, we attribute the weight reduction and improvement in serum liver enzymes of our young patient to carbohydrate restriction although he consumed both a low calorie and low carbohydrate diet. 

Together, the studies reviewed here suggest that LC diets are effective in lowering hepatic steatosis, serum liver enzymes, liver inflammation and fibrosis, and de-novo lipogenesis. However, there are limitations to this review. One major limitation of the studies conducted by Browning et al. [7] and Martens et al. [10] is that they did not specify whether the carbohydrates in the diets were simple or complex. Diets high in simple carbohydrates and low in fiber cause short periods of hyperglycemia, thereby promoting insulin resistance and hepatic steatosis [17]. Complex carbohydrates are less likely to do this [17]. Ryan et al [9] and Fraser et al. [8], however, took this into account by comparing LC diets with a diet high in complex carbohydrates. Their studies suggest that LC diets are superior in reducing hepatic steatosis. Also omitted in all the studies except Ryan et al. [9] was information on fiber, which would be expected to be high in an HC diet if the carbohydrates consist of whole grains and fruit, but low in an HC diet if the carbohydrates consisted of refined sugars and grains. Fiber could therefore constitute a confounding factor that has not been adequately accounted for. 

Much of the data in the studies stemmed from questionnaires, surveys, or interviews, the information therefore could have been affected by recall bias. Randomized clinical trials eliminate recall bias; however, they suffer from small study population sizes and are not immune to patient noncompliance to diet. This is warranted mainly due to a lack of resources to provide customized plans and reviews with dieticians, as well as meal preparation in some of these studies. Small sample sizes will exacerbate any other biases present in the study, such as noncompliance, as observed by Tendler et al. [14]. Meta-analyses of multiple studies with small sample sizes could aid in addressing this. 

NAFLD prevails between 9-45% in South Asia and in the span of two decades, it spiked to an epidemic proportion of 30% in the younger South Asians [18]. Limited data is available on dietary intervention in the South Asian population with NAFLD, therefore our case report stands unique in terms of implementing one such intervention in a young, South Asian male with NAFLD. No such case report has been presented to the best of our knowledge. Despite the same pathophysiology and risk factors (central obesity, visceral fat, Metabolic syndrome, type II DM, etc), South Asians, Hispanics, East Asians are more prone to developing NAFLD compared to other ethnicities. With a later age (50-55yrs) of onset of the disease among the Western population, the mean BMI of the western population (30 to 35 kg/m2) with NAFLD remains greater than South Asians (29 kg/m2 in India and 27 kg/m2 in Sri Lanka).

Future studies could take advantage of diet programs that promote LC diets for weight loss, such as the Atkins Program or the Zone Diet. These programs have already been providing customized diet plans and reviews with dieticians. The researcher’s role would be limited to the administration of tests for NAFLD before and after the patient completes the diet. In addition, these diets emphasize the use of low glycemic index vegetables, to help compensate for the fiber that would otherwise be missing in an LC diet. Collaborating with popular diet programs emphasizing LC diets could address the lack of resources and participants needed to conduct randomized clinical trials on the efficacy of LC diets for NAFLD. In light of the success of other research-based on “crowdsourcing” of data (e.g., from 23&Me and The American Gut Project), a similar approach in collaboration with a major diet program could yield large samples of data to shed light on the potential benefits of LC diets for NAFLD.

Consumption of LC diets have risks associated with reduced dietary fiber intake leading to constipation and negative effects on the microbiome [17]. High levels of protein in LC diets raise the risk of renal dysfunction [17]. The development of osteopenia and osteoporosis is also associated with an LC diet [17]. Future epidemiological studies should investigate the risk-benefit ratio of an LC diet in patients with NAFLD.

## Conclusions

The above-described case and reviewed studies support the hypothesis that carbohydrate restriction could reduce hepatic fat accumulation, liver inflammation, and serum liver enzyme levels in those with NAFLD. Comparison of ADA, LGI, and MM diets, in particular, demonstrate the impact on serum ALT. MM diet with a lower carbohydrate percentage (35%) reduced serum ALT more as compared to ADA and LGI diets with 50-55% carbohydrates. The study also demonstrates that carbohydrate restriction is equally effective in South Asians as in other races therefore, emphasis on cutting down carbohydrates may prove to be helpful in managing the disease burden that is prevalent in South Asia. As low carbohydrate diets do have associated risks, an analysis of the risk-benefit ratio of an LC diet should be further investigated. 

## References

[1] York LW, Puthalapattu S, Wu GY (2009). Nonalcoholic fatty liver disease and low-carbohydrate diets. Annu Rev Nutr.

[2] Simmons RK, Alberti KG, Gale EA (2010). The metabolic syndrome: useful concept or clinical tool? Report of a WHO Expert Consultation. Diabetologia.

[3] Alberti KG, Eckel RH, Grundy SM (2009). Harmonizing the metabolic syndrome: a joint interim statement of the International Diabetes Federation Task Force on Epidemiology and Prevention; National Heart, Lung, and Blood Institute; American Heart Association; World Heart Federation; International Atherosclerosis Society; and International Association for the Study of Obesity. Circulation.

[4] Yki-Järvinen H (2014). Non-alcoholic fatty liver disease as a cause and a consequence of metabolic syndrome. The. Lancet Diabetes & Endocrinology.

[5] Johnson NA, Keating SE, George J (2012). Exercise and the liver: implications for therapy in fatty liver disorders. Semin Liver Dis.

[6] Kotronen A, Juurinen L, Tiikkainen M, Vehkavaara S, Yki-Järvinen H (2008). Increased liver fat, impaired insulin clearance, and hepatic and adipose tissue insulin resistance in type 2 diabetes. Gastroenterology.

[7] Browning JD, Baker JA, Rogers T, Davis J, Satapati S, Burgess SC (2011). Short-term weight loss and hepatic triglyceride reduction: evidence of a metabolic advantage with dietary carbohydrate restriction. Am J Clin Nutr.

[8] Fraser A, Abel R, Lawlor DA, Fraser D, Elhayany A (2008). A modified Mediterranean diet is associated with the greatest reduction in alanine aminotransferase levels in obese type 2 diabetes patients: results of a quasi-randomised controlled trial. Diabetologia.

[9] Ryan MC, Abbasi F, Lamendola C, Carter S, McLaughlin TL (2007). Serum alanine aminotransferase levels decrease further with carbohydrate than fat restriction in insulin-resistant adults. Diabetes Care.

[10] Martens EA, Gatta-Cherifi B, Gonnissen HK, Westerterp-Plantenga MS (2014). The potential of a high protein-low carbohydrate diet to preserve intrahepatic triglyceride content in healthy humans. PLoS One.

[11] Toshimitsu K, Matsuura B, Ohkubo I (2007). Dietary habits and nutrient intake in non-alcoholic steatohepatitis. Nutrition.

[12] Sevastianova K, Santos A, Kotronen A (2012). Effect of short-term carbohydrate overfeeding and long-term weight loss on liver fat in overweight humans. Am J Clin Nutr.

[13] Goss AM, Dowla S, Pendergrass M (2020). Effects of a carbohydrate-restricted diet on hepatic lipid content in adolescents with non-alcoholic fatty liver disease: A pilot, randomized trial. Pediatr Obes.

[14] Tendler D, Lin S, Yancy WS Jr, Mavropoulos J, Sylvestre P, Rockey DC, Westman EC (2007). The effect of a low-carbohydrate, ketogenic diet on nonalcoholic fatty liver disease: a pilot study. Dig Dis Sci.

[15] Adiels M, Westerbacka J, Soro-Paavonen A (2007). Acute suppression of VLDL1 secretion rate by insulin is associated with hepatic fat content and insulin resistance. Diabetologia.

[16] Della Pepa G, Vetrani C, Lombardi G, Bozzetto L, Annuzzi G, Rivellese AA (2017). Isocaloric Dietary Changes and Non-Alcoholic Fatty Liver Disease in High Cardiometabolic Risk Individuals. Nutrients.

[17] Czyżewska-Majchrzak Ł, Grzelak T, Kramkowska M, Czyżewska K, Witmanowski H (2014). The use of low-carbohydrate diet in type 2 diabetes - benefits and risks. Ann Agric Environ Med.

[18] Pati GK, Singh SP (2016). Nonalcoholic Fatty Liver Disease in South Asia. Euroasian J Hepatogastroenterol.

